# Case report: inability to achieve a therapeutic dose of tacrolimus in a pediatric allogeneic stem cell transplant patient after generic substitution

**DOI:** 10.1186/2050-6511-15-69

**Published:** 2014-12-03

**Authors:** Ashraf G Madian, Arun Panigrahi, Minoli A Perera, Navin Pinto

**Affiliations:** Committee of Clinical Pharmacology and Pharmacogenomics, The University of Chicago, Chicago, IL USA

**Keywords:** Tacrolimus, Generic, Children

## Abstract

**Background:**

Tacrolimus is an immunosuppressive drug that is used to lower the activity of the patient’s immune system to prevent organ rejection. Unfortunately, there is limited data regarding the therapeutic equivalency of generic tacrolimus formulations especially in children. We report the case of a pediatric patient having an inability to achieve a therapeutic trough level for tacrolimus after conversion from brand name to the generic formulation.

**Case presentation:**

A 17-month-old male patient diagnosed with T-cell acute lymphoblastic leukemia underwent allogeneic stem cell transplantation. The patient initially received intravenous (IV) tacrolimus for graft-versus-host disease (GVHD) prophylaxis and achieved therapeutic levels. The patient was then switched to an oral brand formulation of tacrolimus, and was able to maintain trough levels within the therapeutic range. After being discharged, the patient received the generic formulation of tacrolimus from an outside pharmacy and the care team was unable to reach therapeutic levels despite multiple dose escalations. Returning to brand name tacrolimus resulted in prompt achievement of therapeutic levels.

**Conclusions:**

A likely etiology for the inability to achieve therapeutic trough levels in this patient is the change in formulation from brand formulation to generic version. Other factors including drug-drug interaction, preparation of the medication by a different pharmacy, drug-food interaction and genetic factors were also considered. Physicians and pharmacists must be aware of the inability to achieve targeted therapeutic concentrations of tacrolimus resulting from the conversion of brand name to the generic formulation until these generic formulations are tested in clinical trials in a pediatric population.

## Background

Tacrolimus is a commonly used calcineurin inhibitor used to induce immunosuppression and prevent graft-versus-host disease as well as rejection in patients receiving both hematopoietic and solid organ transplantation [1–3]. When approved in 1994, it was marketed under the brand name Prograf®. The first generic version was approved by the FDA in 2009 [4]. In practice there is a sentiment among physicians and patients that generic immunosuppressants differ in efficacy from their brand versions [5]. The FDA uses a simplified process for the approval of generic drugs called an abbreviated new drug application (ANDA) [1]. In that process the generic drug is tested only in healthy volunteers with ages between 24–36 years old. The FDA defines bioequivalence as “the absence of a significant difference in the rate and extent to which the active ingredient or active moiety in pharmaceutical equivalents or pharmaceutical alternatives becomes available at the site of drug action when administered at the same molar dose under similar conditions in an appropriately designed study.” Currently, the FDA does not place immunosuppressants within a special category when evaluating the bioequivalency between generic and brand name drugs [5].

Although there are 5 generic formulations of tacrolimus currently available in the U.S. [6], there is limited data to confirm their therapeutic equivalency. In this case report we describe a 17-month-old boy with T-cell acute lymphoblastic leukemia who underwent a matched unrelated allogeneic stem cell transplant approximately two months prior. He was initially started on tacrolimus (i.v.) at the time of transplant for GVHD prophylaxis, and after changing to oral brand formulation in the hospital he was still in the therapeutic range. Once he was discharged, he received generic tacrolimus from an outside pharmacy, and was found to be sub-therapeutic despite escalating doses of medication.

## Case presentation

**Figure 1:**
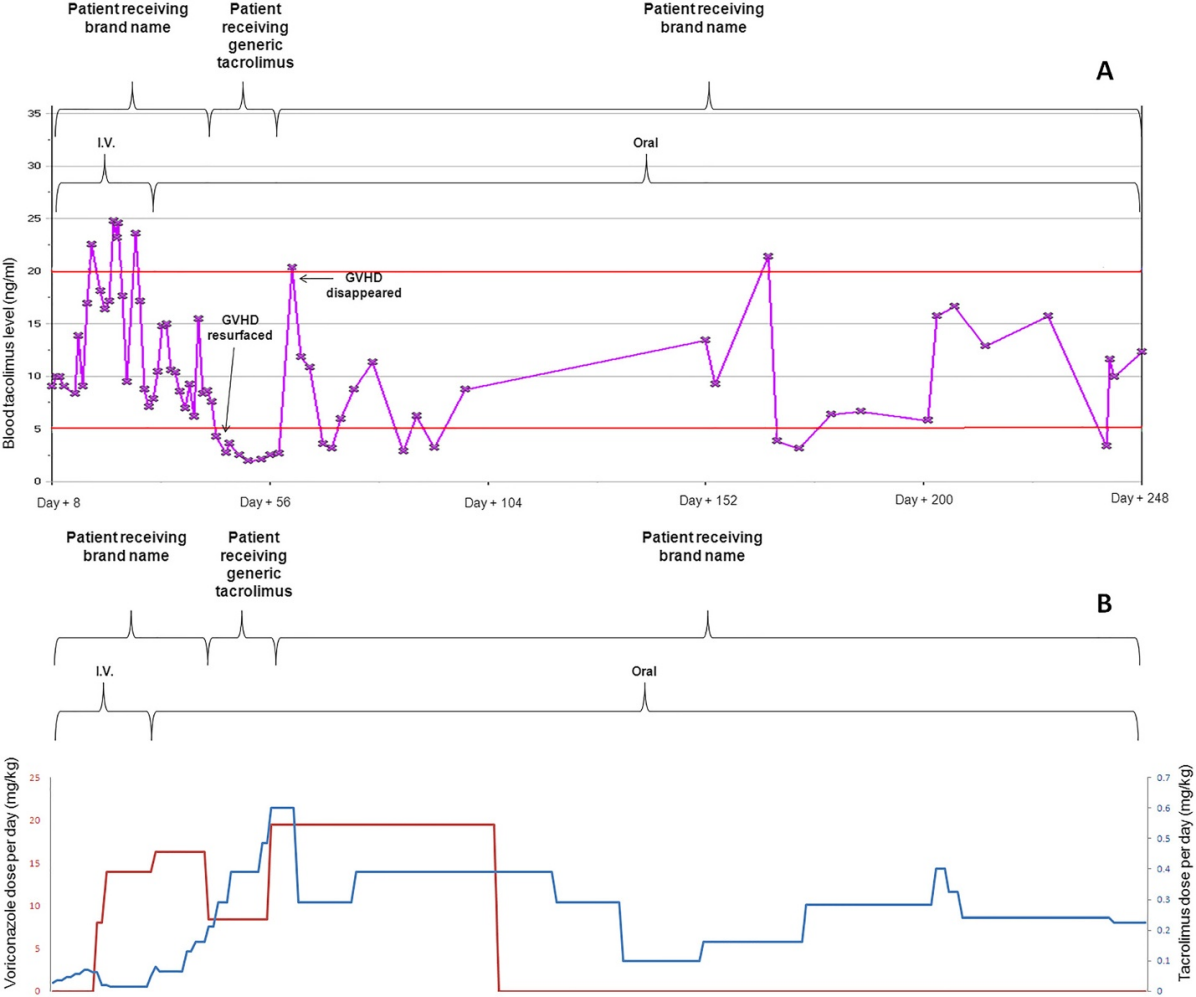
**Tacrolimus blood levels compared to its daily dose. (A)** Blood levels of tacrolimus when patient administered brand or generic version. Day 0 was when patient received matched unrelated allogeneic stem cell transplant. X represents the patient’s blood tacrolimus levels (ng/ml). Red lines represent the upper and lower limits of tacrolimus therapeutic trough level. (GVHD): graft-versus-host disease. **(B)** Tacrolimus dose per day (mg/kg) and voriconazole dose per day (mg/kg) for the same time interval.

A 17-month-old male patient was diagnosed with T-cell acute lymphoblastic leukemia at 10 months of life, when he was noted to have a white blood cell count of 950,000 with peripheral leukemic blasts as well as systemic symptoms. Subsequently he received multiple courses of chemotherapy, and then underwent a matched unrelated allogeneic stem cell transplant at the age of 15 months. A combination of Busulfan, Fludarabine and Alemtuzumab were utilized for myeloablation prior to allogeneic stem cell transplantation from a matched unrelated donor. Subsequently he was initially started on IV tacrolimus (0.033 mg/kg) for GVHD prophylaxis, and achieved therapeutic levels (Figure 1). Approximately one month after transplant in anticipation of being discharged, the patient was switched to an oral brand name formulation of tacrolimus (Prograf®), and was able to maintain trough levels in the prescribed therapeutic window (Figure 1). The patient was discharged approximately one week later with generic tacrolimus suspension dosed at 0.15 mg/kg PO twice daily which was compounded at an outside pharmacy. Subsequently, he was unable to reach therapeutic levels despite multiple escalations in dosage to a maximum dosage of 0.31 mg/kg PO twice daily (Figure 1). Also during this time the patient’s dose of Voriconazole was reduced from 16.26 mg/kg (therapeutic dosage) to 8.46 mg/kg PO daily (expected prophylactic dosage). During this period when his doses were escalated due to inadequate trough levels, multiple investigations were made, and the pharmacist compounding the medication was contacted. According to the outside pharmacy, the pharmacist compounded the medication in a similar fashion to the inpatient pharmacy and the solvents used were the same. The compounding in the inpatient and outside pharmacy followed a straightforward procedure involving mixing the contents of 6 tacrolimus capsules (5 mg each) with 30 mls of Syrup and 30 mls of oral suspending vehicle. Trough levels were drawn at appropriate times, and the family was compliant with the medication.

Initially after transplant the patient manifested evidence of skin GVHD with mild skin erythema; topical steroids were initiated two weeks prior to discharge and were continued as an outpatient. The child’s skin GVHD showed marked improvement with topical steroids yet began to flare a few weeks later when he presented to the clinical pharmacology service for inability to reach a therapeutic level for tacrolimus. Tacrolimus is one of the primary agents used to induce immunosuppression and combat GVHD in bone marrow transplant patients; hence the patient’s resurgence of skin GVHD is likely further manifestation of sub-therapeutic tacrolimus levels.

At the time of the initial encounter for skin GVHD and subtherapeutic tacrolimus levels the patient was taking the following medications: acetaminophen (15 mg/kg by oral route every 6 hours as needed for pain for 30 doses), diphenhydramine (1 mg/kg by oral route every 6 hours as needed), famotidine (0.53 mg/kg by oral route twice daily), hydrocortisone 0.5% topical ointment (1 application by topical route twice daily), ondansetron (0.15 mg/kg by oral route every 8 hours as needed for nausea/vomiting), sulfamethoxazole-trimethoprim (13.3 mg/kg/2.6 mg/kg) by oral route twice daily on Monday, Tuesday, Wednesday), valacyclovir (29 mg/kg by oral route every 8 hours), voriconazole (oral suspension 10 mg/kg by oral route twice daily), and multivitamins. Patient had an appropriate response to opiates (including codeine) and other medications per the caregivers. Patient did not have any adverse outcomes with surgery and anesthesia.

Review of systems at the initial encounter indicated the patient was irritable due to pruritus. The patient had a generalized rash that caused him to wake at night and necessitated the use of diphenhydramine for symptomatic relief. He also had loose stools yet normal number of bowel movements daily, and was tolerating his diet appropriately. He did not have fever, or a change in appetite or activity. Physical exam showed a fine erythematous rash scattered on face and extremities. Excoriations were present on the lower back and extremities as well. The clinical pharmacology service was consulted at this time to evaluate the etiology of the patient’s inability to reach therapeutic trough levels of tacrolimus.

## Discussion

In clinical practice we utilize trough level concentration as a measure of the exposure to tacrolimus. A recent study showed that trough level concentrations can be used as a reliable surrogate measure for area under the curve (AUC) for patients in normal clinical practice receiving generic tacrolimus [7].

Several factors, which may have contributed to the subtherapeutic trough levels upon conversion to generic formulation, were considered. For example, although the outside pharmacy claimed that it prepared the medication in a similar way as the hospital pharmacy, it still remains a possibility that this pharmacy had less experience in compounding narrow therapeutic index drugs (NTI) which may have contributed to the difference in drug levels. Additionally, it’s known that voriconazole can inhibit CYP3A4, decreasing the metabolism of tacrolimus and increasing its blood concentration [8]. As shown in Figure 1, the patient’s dose was reduced from 16.26 mg/kg (therapeutic dosage) to 8.46 mg/kg PO daily (expected prophylactic dosage) that may have resulted in increasing the metabolism of tacrolimus and decreasing its concentration in the plasma. The dose of voriconazole was increased back to 19.5 mg/kg/day at the same time of switching back to the brand name tacrolimus and the rise of blood tacrolimus levels. Therefore the change in voriconazole dose could have contributed to the change in blood tacrolimus levels but because the patient maintained therapeutic drug concentrations after discontinuing voriconazole on day +106, the clinical pharmacology consultation team hypothesized that drug-drug interactions did not fully contribute to the observed drop in tacrolimus levels.

Additionally, it’s unlikely that famotidine administration caused the environment in the stomach to be alkaline and the patient was given it both while in the hospital and after being discharged. Moreover, clinical literature has been inconclusive regarding the impact of antacids on immunosuppresants such as tacrolimus [9]. Moreover, the prevention of tacrolimus absorption due to the concomitant administration of iron or other vitamins is not supported by any clinical literature. In addition to that, the patient received the same iron and vitamins while he was in the hospital. Finally, the other drugs he administered (Acetoaminophen, diphenhydramine, ondansetron, sulfamethoxazole-trimethoprim and valacyclovir) were not reported to interact with tacrolimus. Furthermore, the patient was given these drugs both while he was in the hospital and after being discharged. Finally, he didn’t have any food changes that may have contributed to the changes in bioavailability between the two products when they were given orally.

Pharmacogenetic factors were unlikely to have contributed. The CYP 3A5*1 allele has been implicated in lower tacrolimus trough levels than patients with CYP3A5*3 [10]. This allelic variant, though important, is unlikely to play a critical role for our patient, as he was able to maintain therapeutic trough levels with appropriate dosage of tacrolimus.

The conversion from brand to generic formulation could have contributed to the inability to achieve therapeutic levels of tacrolimus. In general, studying the effect of switching to generic tacrolimus in pediatric patients is not well explored. The only available study that evaluated switching to generic tacrolimus in children was done in renal transplant patients [4]. In that study, both trough and serum creatinine levels were retrospectively analyzed for four patients (with ages range between 8–22 years old). Although trough levels were generally comparable before and after switching from brand name to generic tacrolimus, interindividual differences existed. Serum creatinine levels were identical pre- and post- switch in three of the four patients. The fourth patients suffered from acute rejection immediately after switching to the generic formulation. This was accompanied by a dramatic increase in the patient’s serum creatinine level.

Contradictory results exist regarding the clinical equivalency of brand name tacrolimus formulations to their generic versions in the adult population. Despite the aforementioned study that showed that brand name tacrolimus and generic formulations may not be bioequivalent, other studies have showed that they are bioequivalent. For example, one study examined the efficacy and safety of the generic oral capsules of tacrolimus (TacroBell®) in de novo renal transplantation [11]. The study recruited ninety-six renal transplant recipients from 9 transplantation centers in South Korea. In general, the acute rejection and graft survival rates were comparable to brand name treatment. One unresolved issue with this study is that it was carried out in low risk populations with only short term follow up. Another recent study evaluated seventy conversions, of brand name tacrolimus to the generic tacrolimus (Sandoz), from four centers from patients after kidney, liver or multiorgan transplant [12]. This study showed that trough levels and dosage needed are similar between brand name tacrolimus and its generic formulation. Furthermore, a retrospective analysis of the electronic records and clinical databases for 234 clinically stable adult transplant recipients (renal, liver, and heart) whose tacrolimus was converted from brand name to a generic formulation recently occurred [13]. Trough levels were generally comparable between the two formulations. No deaths or acute rejections were reported but thirty-six patients required dose titration. Another open-label, multicenter pilot study in South Korea evaluated 57 patients receiving generic tacrolimus and corticosteroids after liver transplantation [14]. This group of patients was then compared to another retrospectively matched control group consisting of living donor liver transplant recipients at another center who received brand name tacrolimus. Adverse events were generally comparable between the two patient populations and no patients died during this study. In addition to that, a multicenter crossover pharmacokinetic study in which patients would receive both the generic and brand name formulation for 14 days and then crossover revealed that Sandoz generic tacrolimus has a similar pharmacokinetic profile to Prograf® in kidney transplant patients [15]. Finally, a recent prospective study showed that stable kidney transplant recipients can be converted to Sandoz generic tacrolimus by closely monitoring both plasma creatinine levels and trough concentration of tacrolimus [16].

For our patient, we recommended that the patient return to brand name formulation, after which the patient had therapeutic tacrolimus levels as well as resolution of his GVHD flare.

There are a number of reasons why the current bioequivalence studies may be inadequate for the approval of generic immunosuppressants [17]. First, studies in healthy subjects may not be extrapolated to transplant recipients. Second, steady state conditions may not be represented by single dose studies. Third, the allowance of the confidence interval to be (80%–125%) may be too variable for drugs with a narrow therapeutic index such as immunosuppressants. Finally, children are excluded from these bioequivalence studies. For this reason the American Society of Transplantation strongly supported studies to demonstrate bioequivalence in potentially at risk patients, especially children [18].

## Conclusions

The fact that previous studies regarding the bioequivalency of generic and brand name tacrolimus were almost exclusively done in adult human highlights the importance of testing them in pediatric patients as well as those who have undergone allogeneic stem cell transplantation. Results from adult case studies cannot be extrapolated for children. Until the use of various generic formulations of tacrolimus can be evaluated in a large randomized clinical trial in children, physician and pharmacists must be aware of possible adverse events following a conversion from brand name to the generic form of tacolimus.

## Consent

Written informed consent was obtained from the patient’s guardian for publication of this case report.
